# Exosomes Secreted from Amniotic Membrane Contribute to Its Anti-Fibrotic Activity

**DOI:** 10.3390/ijms22042055

**Published:** 2021-02-19

**Authors:** Yong Mao, Vimal Jacob, Amit Singal, Shunyao Lei, Min Sung Park, Mariana R.N. Lima, Chaoyang Li, Sandeep Dhall, Malathi Sathyamoorthy, Joachim Kohn

**Affiliations:** 1 Laboratory for Biomaterials Research, Department of Chemistry and Chemical Biology, Rutgers University, 145 Bevier Rd., Piscataway, NJ 08854, USA; aamitsingall@gmail.com (A.S.); shunyaolei0817@gmail.com (S.L.); mrr190@scarletmail.rutgers.edu (M.R.N.L.); kohn@dls.rutgers.edu (J.K.); 2 Smith & Nephew, 7015 Albert Einstein Drive, Columbia, MD 21046, USA; vimal.jacob@smith-nephew.com (V.J.); minsung.park@smith-nephew.com (M.S.P.); zhaoyli@gmail.com (C.L.); sandeep.dhall@smith-nephew.com (S.D.); msathyam39@hotmail.com (M.S.)

**Keywords:** amniotic membrane, exosomes, LX-2, anti-fibrotic activity, collagen I

## Abstract

Amniotic membranes (AM) have anti-fibrotic activity. Exosomes (nano-sized vesicles) function as conduits for intercellular transfer and contain all the necessary components to induce the resolution of fibrosis. In this study, we tested the hypothesis that the anti-fibrotic activity of AM is mediated by exosomes. AM-derived exosomes or amniotic stromal cell-derived exosomes were isolated and characterized. Anti-fibrotic activity of exosomes was evaluated using human hepatic stellate cells (LX-2), an in vitro model of fibrosis. Exosomes isolated from AM tissue-conditioned media had an average size of 75 nm. Exosomes significantly inhibited the proliferation of TGFβ1-activated LX-2 but had no effect on the proliferation of non-activated LX-2 cells. Exosomes also reduced the migration of LX-2 in a scratch wound assay. Furthermore, exosomes reduced the gene expression of pro-fibrotic markers such as COL1A1, ACTA, and TGFβ1 in LX-2 cells. Interestingly, exosomes isolated from AM tissue under hypoxic conditions seemed to show a stronger anti-fibrotic activity than exosomes isolated from tissue under normoxic conditions. Exosomes released by in vitro cultured AM stromal cells were smaller in size compared with tissue exosomes and also showed anti-fibrotic activity on LX-2 cells. In conclusion, AM-tissue-released exosomes contribute to the anti-fibrotic activity of AM. This is the first report of isolation, characterization, and functional evaluation of exosomes derived from amniotic tissues with the direct comparison between tissue-derived exosomes and cultured cell-derived exosomes.

## 1. Introduction

Fibrosis is an excess accumulation of extracellular matrix components in tissues or organs, which results in the disruption of normal functions of affected tissues or organs [1]. Fibrosis can develop in nearly every part of the body and leads to organ failure and death in a variety of chronic diseases [1,2]. The mechanism of fibrosis has been well studied. Among many activating factors, transforming growth factor-β1 (TGF-β1) has been shown to play a central role in regulating the initiation and progression of fibrosis [3]. Liver fibrosis is a characteristic of most chronic liver diseases [4,5,6]. Hepatic stellate cell (HSC) activation by TGF-β1 has been attributed to the excess deposition of type I collagens in the liver, which subsequently leads to fibrosis [7,8]. HSC therefore has become a potential target for anti-fibrosis therapies [9,10].

Amniotic membrane (AM) has been used in treating burns and chronic wounds for over a century [11,12]. In recent years, processed and preserved AM has been developed into ready-to-use wound therapies [13]. The clinical benefit of AM in treating various wounds, including chronic wounds, has been established and recognized [14]. Fibrosis is a pathological process due to a deregulated wound-healing process in response to chronic tissue injury and/or inflammation [1]. Exploring the benefits of AM in treating fibrotic diseases has become one of the major focuses of anti-fibrosis research. Anti-fibrotic activity of fresh AM has been reported in animal models [15,16,17,18]. AM contains multiple anti-fibrotic growth factors and cytokines. Mesenchymal stem cells (stromal cells) and epithelial cells isolated from AM show anti-fibrotic activity [19,20]. Anti-fibrotic activity of preserved viable AM has also been demonstrated in vitro [21]. The conditioned media from viable AM showed anti-fibrotic activity on human lung fibrotic fibroblasts and suggested viable AM releases anti-fibrotic factors. However, the source and nature of such anti-fibrotic factors remain to be identified [21].

Exosomes are 30–100 nm endosome-derived vesicles released from a wide range of cells. Exosomes transport molecular signals between cells and regulate the phenotype of the target cell locally or remotely [22,23]. Exosomes have specific characteristics and a biogenesis pathway different from other extracellular vesicles such as microvesicles (100–1000 nm) and apoptotic bodies (50–5000 nm) [24,25,26]. Biogenesis of exosomes begins with early endosomes by the endocytosis of transmembrane proteins [26]. Once sorted to late endosomes, an endosomal sorting complex required for transport (ESCRT) recruits proteins, nucleic acids, and lipids to late endosomes. ESCRT then mediates the budding of late endosomes and formation of the intraluminal vesicles inside of the multivesicular body (MVB). During this process, intraluminal vesicles are able to recruit cargos specific to the origin cells. Some intraluminal vesicles can be released to extracellular space through exocytosis and become exosomes [27]. Moreover, all exosomes are enriched with transmembrane tetraspanins (CD9, CD63, and CD81) that play an important role in the biogenesis of exosomes and the uptake of exosomes by the target cells [28]. While sharing common characteristics, exosomes released by a given cell type can have distinct composition due to the specific cargoes (i.e., proteins, miRNA, and lipids) and confer distinct functions to target cells [29]. Not only does the origin of cells influence the functionality of exosomes, but the physiological state of cells also affects the production and activity of exosomes. Stresses such as hypoxia and oxidative stress have been shown to alter the characteristics of exosomes [23,30]. Exosomes released from cells cultured under hypoxic conditions often have a higher CD63 presence and contain selective protein and miRNA compared with exosomes released by the same cells under normoxic conditions [31,32,33].

Exosomes from placental tissues have been isolated either from body fluids (plasma and urine) of pregnant women or from in vitro cultured placental cells (epithelial cells and stromal cells derived from AM) [23,30,34,35,36,37,38,39,40]. Exosomes isolated from placental tissues are also enriched with tetraspanins. The average sizes of exosomes isolated from AM epithelial cells and AM stromal cells were reported to be in the range of 30–100 nm [30,40,41]. Stress conditions influence the production and characteristics of exosomes from AM epithelial cells and AM stromal cells [23,30].

While exosomes released from in vitro cultured cells have been extensively studied, exosomes produced directly from tissues have not been widely explored. Since exosomes’ formation and activity are sensitive to cell physiological states, one may postulate that a native tissue microenvironment may also influence the release and activity of exosomes. To understand if amniotic membrane tissue produces and releases exosomes to its environment, and if it does, whether such exosomes have anti-fibrotic activity, exosomes released from AM tissues were isolated and characterized in this study. The effects of physiological conditions (hypoxia versus normoxia or native microenvironment versus in vitro cell culturing) on the production of exosomes were evaluated by comparing exosomes produced by tissues under normoxic condition with those under hypoxic conditions and comparing exosomes released by AM tissues with exosomes isolated from in vitro cultured AM stromal cells. Exosomes from AM-derived cells have been shown to have anti-fibrotic activity [42,43,44]. The anti-fibrotic activity of AM tissue-derived exosomes was evaluated in human hepatic stellate cells, LX-2, which is a widely used model cell line for studying the fibrotic process [45]. We showed that exosomes released by AM tissue shared the characteristics of exosomes released by cultured AM stromal cells but with some distinctions. We also showed that AM tissues respond to hypoxic conditions and release exosomes with enhanced activity. Exosomes from tissue-released or cell-released exosomes showed anti-fibrotic activity in LX-2 cells. This observation suggests that tissue-released exosomes may be a source for the anti-fibrotic activity observed in AM tissues [21]. To our knowledge, our study is the first report on the isolation and characterization of exosomes released by viable amniotic membrane tissue. Exosomes released from cultured cells have been evaluated as an emerging anti-fibrosis therapy [46], and our study showed that exosomes released by AM tissues also have the potential to be developed into a cell-free anti-fibrosis therapy.

## 2. Results

### 2.1. Isolation and Characterization of Exosomes from AM Tissue

**Figure 1:**
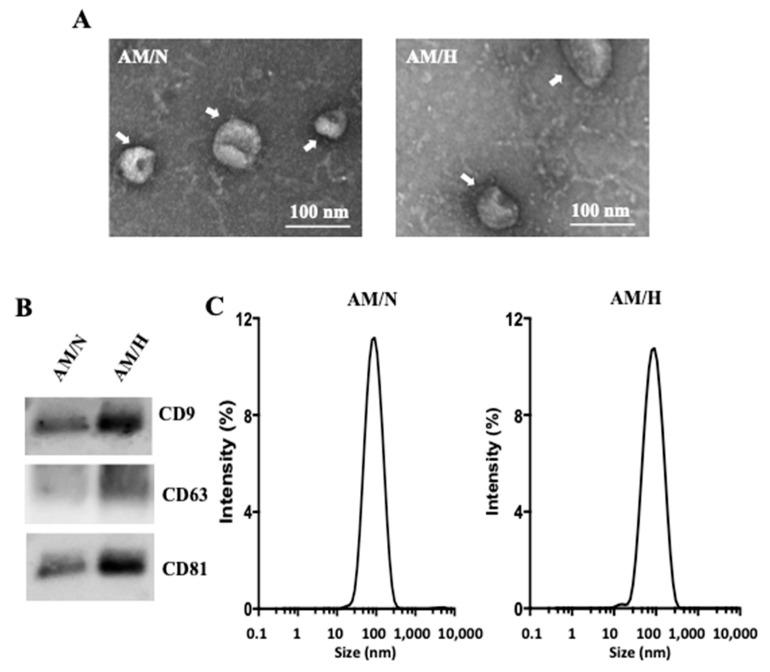
Characterization of exosomes isolated from amniotic membrane (AM). Exosomes were isolated from conditioned media prepared from AM under normoxic (AM/N) or hypoxic (AM/H) conditions as described in the Methods. Exosomes were examined by transmission electron microscopy (**A**). Representative exosomes are indicated by white arrows. Scale bar = 100 nm. The presence of tetraspanins in exosomes was determined by SDS-PAGE followed by Western blot using antibodies against human CD9, CD63, and CD81 (**B**). The particle size of exosomes was analyzed using dynamic light scattering. The size distributions were graphed against the percentage of intensity (**C**).

A sequential ultracentrifugation and filtration method were used to isolate exosomes from conditioned media prepared from AM tissues under normoxic (AM/N) or hypoxic (AM/H) conditions. The exosomes showed spherical morphology under transmission electron microscopy (Figure 1A). The presence of the characteristic markers of exosomes, tetraspanins (CD9, CD81, and CD63) was confirmed by Western blot (Figure 1B). In addition, the absence of a microvesicle marker, CD40, was also confirmed (data not shown). With the same protein loading, AM/H exosomes showed higher level of CD9, CD81, and CD63 and suggested that AM/H exosomes are likely more enriched with tetraspanins. The sizes of exosomes from different AM donors were also analyzed using dynamic light scattering (Figure 1C). Results all showed a single peak distribution and had an average size of 75.4 ± 1.4 nm (*n* = 3) for AM/N and 73.8 ± 1.1 nm (*n* = 3) for AM/H. The size distribution was consistent with the observation with TEM analysis. There was no obvious difference in size between the exosomes from AM/N or AM/H. Altogether, the isolated vesicle fraction by sequential ultracentrifugation showed the prominent characteristics of exosomes. Quantified by BCA assay, the yields of exosomes from the AM tissues (area in cm^2^) under normoxic or hypoxic were 2.0 μg/cm^2^ for AM/N and 2.3 μg/cm^2^ for AM/H.

### 2.2. Effect of AM Exosomes on the Proliferation of LX-2 Cells

**Figure 2:**
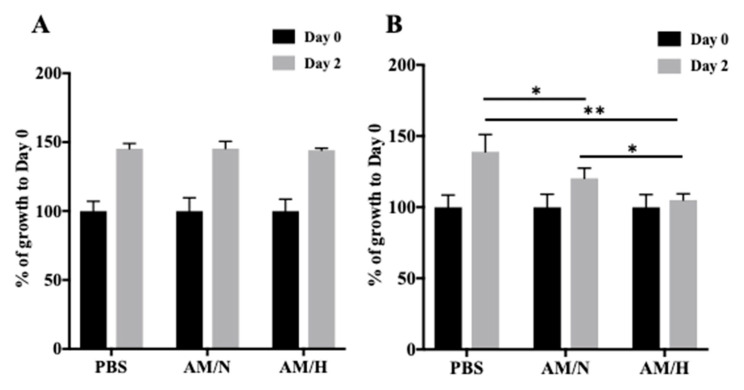
The effect of AM exosomes on the proliferation of LX-2 cells. LX-2 cells were activated with 4 ng/mL of TGFβ-1 for 3 h. Exosomes isolated from AM under normoxic condition (AM/N) or exosomes isolated from AM under hypoxic condition (AM/H) at 5 μg/mL were added to non-activated (**A**) or activated LX-2 cells (**B**) and incubated for 2 days. % of growth on Day 2 is expressed as % of increase in fluorescent intensity over that of Day 0. Data shown are mean ± SD (*n* = 4). * *p* < 0.05, ** *p* < 0.01.

Recent findings have shown the anti-fibrotic potential of exosomes isolated from in vitro cultured amniotic membrane-derived cells [42,43,44]. To understand if the exosomes isolated from AM tissues have anti-fibrotic activity, we used the LX-2 cell line, which is a widely used human hepatic stellate cell line for studying fibrotic responses [45]. We first examined the effect of AM exosomes on LX-2 cell proliferation. As shown in Figure 2A, the presence of exosomes (AM/N or AM/H) did not affect the growth of LX-2 (Figure 2A). The proliferation of LX-2 in the presence or absence of exosomes was also analyzed by Click-iT^™^ EdU cell proliferation kit for imaging. Cells showed a similar level of proliferation (Supplemental Figure S1A). It has been shown that LX-2 cells can be activated by the treatment of TGF-β1, which induces a transition of LX-2 cells from a quiescent (non-activated) state to a myofibrotic (activated) state which mimics the onset of the fibrosis process [47]. When LX-2 cells were activated by the treatment of 4 ng/mL of TGF-β1, the presence of exosomes inhibited the growth of LX-2 cells (Figure 2B and Supplemental Figure S1B). Interestingly, the AM/H exosomes showed a slightly stronger inhibitory effect on the growth of activated LX-2 cells. These results indicated that exosomes counteract the effect of TGF-β1 on the growth of LX-2 cells.

### 2.3. The Effects of AM Exosomes on the Expression of Fibrotic Markers

**Figure 3:**
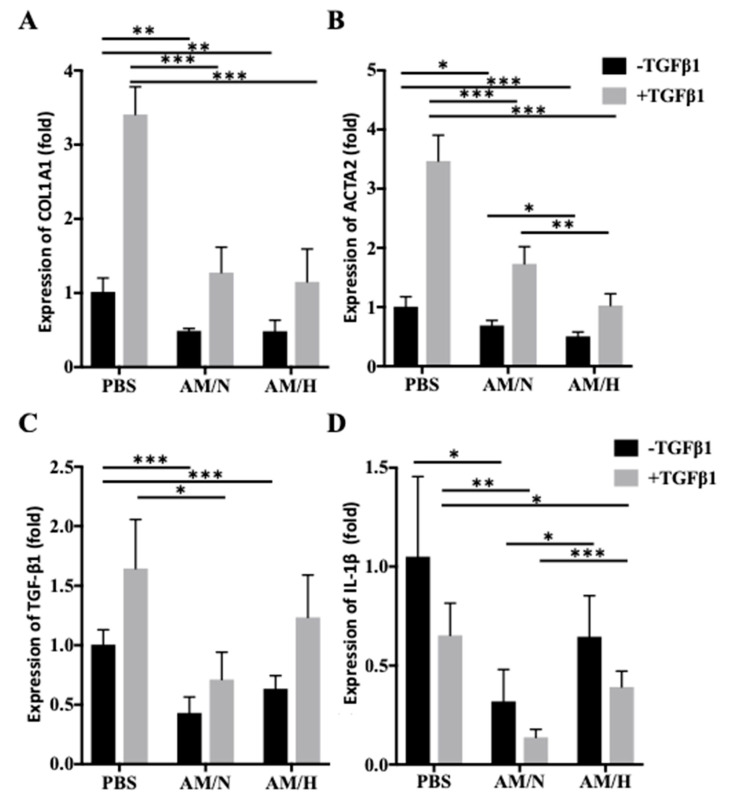
Effect of AM exosomes on the gene expression of fibrotic markers in LX-2 cells. Non-activated (−TGF-β1) and activated (+TGF-β1) LX-2 cells were cultured in the presence or absence of AM exosomes for 2 days. Total RNA was isolated from LX-2 cells, and the relative expression of COL1A1 (**A**), ACTA2 (**B**), TGF-β1 (**C**), and IL-1β (**D**) was quantified by qPCR. The relative expression of each gene in the presence of exosomes was normalized to that of control (PBS (-TGFβ-1)), which was set as 1. Data are presented as mean ± SD (*n* = 4). * *p* < 0.05, ** *p* < 0.01, *** *p* < 0.005.

In order to determine whether the AM exosomes carry anti-fibrotic activities, the expression of fibrotic markers in LX-2 cells was evaluated in the presence or absence of AM exosomes. The expressions of fibrotic markers in non-activated and activated LX-2 cells were also compared (Figure 3). COL1A1 (type I collagen) and ACTA2 (alpha smooth muscle actin) are the most commonly used fibrotic markers [48]. The treatment of TGF-β1 increased the expression of COL1A1 in LX-2 cells (Figure 3A, first and second columns), and the presence of AM exosomes reduced the expression of COL1A1 in both non-activated and TGF-β1-activated LX-2 cells. Strikingly, the treatment of activated LX-2 cells with AM exosomes brought down the gene expression levels to be similar to those of non-activated LX-2 under control conditions (PBS). However, the effect of AM/N versus that of AM/H on LX-2 cells was not significantly different (Figure 3A). Similarly, the treatment with TGF-β1 increased the expression of ACTA2 in LX-2 cells (Figure 3B, first and second columns). The presence of AM exosomes reduced the expression of ACTA2 in non-activated or TGF-β1-activated LX-2 cells. More interestingly, AM/H exosomes demonstrated a stronger inhibitory effect on the expression of ACTA2 in LX-2 compared with AM/N exosomes. The expression of another fibrotic marker, TGF-β1, adopted a similar pattern to that of COL1A1 (Figure 3C). The role of IL-1β in fibrosis seems to be less unified. Both pro-fibrotic and anti-fibrotic activity of IL-1β have been reported [49]. Activation of LX-2 by TGF-β1 did not increase the expression of IL-1β (Figure 3D). Instead, the activated cells showed a decreasing trend, and the presence of AM exosomes decreased the expression of IL-1β even further. Unlike its effect on the expression of ACAT2, AM/N showed a stronger inhibitory effect on the expression of IL-1β than that of AM/H. These results suggested the role of AM exosomes in reducing the expression of pro-fibrotic markers in LX-2 cells.

**Figure 4:**
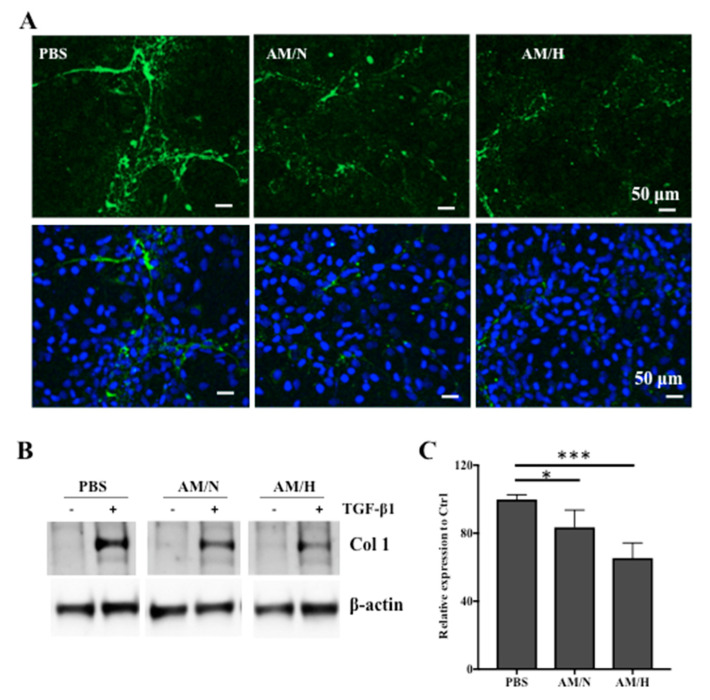
Effect of AM exosomes on the protein expression of type I collagen (Col1). Non-activated LX-2 cells were cultured in the presence (AM/N or AM/H) or absence (PBS) of exosomes for 5 days. Cells were fixed and immunostained with antibody against Col1 ((**A**) upper panel) and merged with Hoechst dye staining ((**A**) lower panel). Representative images are shown (**A**). Scale bar = 50 μm. LX-2 cells were cultured in the presence (AM/N or AM/H) or absence (PBS) of exosomes for 2 days. The expression of Col1 in non-activated (−) and activated (+) LX-2 cells were analyzed by Western blotting using antibodies against Col1 and β-actin. Representative blots are shown (**B**). The relative band intensity of Col1 in the presence of TGF-β1 was quantified using Image J software (**C**). Data are presented as mean ± SD (*n* = 4). * *p* < 0.05, *** *p* < 0.005.

The expression of type I collagen protein (Col1) in LX-2 cells was also evaluated by immunofluorescent staining and Western blotting (Figure 4). As shown in Figure 4A, the deposition of Col1 by LX-2 cells was reduced by the presence of either AM/N or AM/H exosomes. In order to quantify the expression of Col1, non-activated or activated cells were treated with exosomes for 2 days, total protein was analyzed using SDS-PAGE followed by blotting with anti-Col1 (Figure 4B). The treatment of TGFβ-1 induced the expression of Col1 significantly. In the activated LX-2 cells, the treatment of exosomes reduced the expression of Col1 (Figure 4C). These results confirmed that AM exosomes inhibited the expression of pro-fibrotic type I collagen at both gene and protein levels.

### 2.4. The Presence of AM Exosomes Reduced the Migration of LX-2

**Figure 5:**
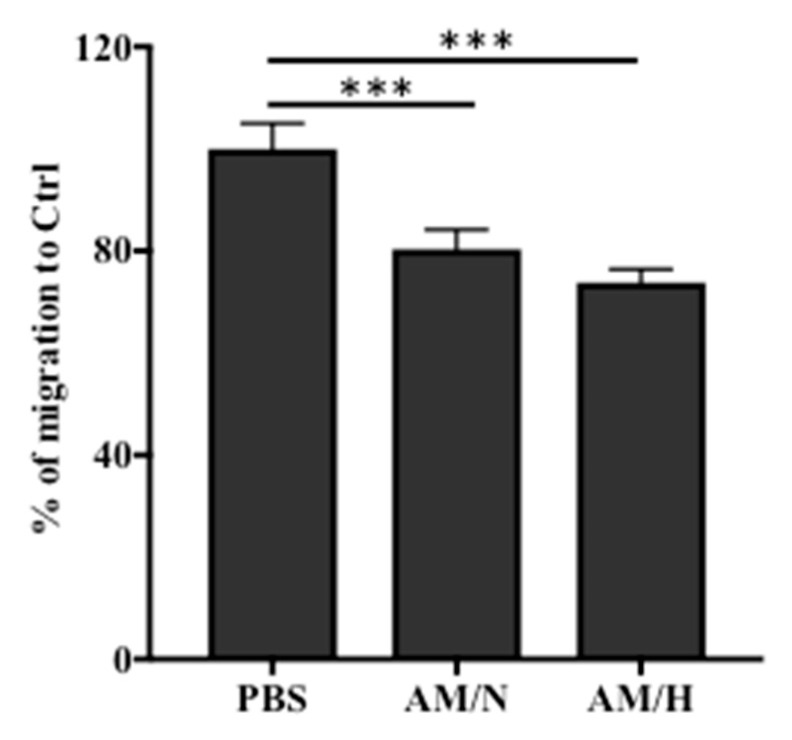
Effect of AM exosomes on the migration of LX-2 cells in a scratch wound assay. Non-activated LX-2 cells were cultured to confluence. A scratch wound was created, and images were captured at time 0. After incubation with PBS or exosomes for 24 h, the wound areas were imaged. The migration of LX-2 cells was expressed as a percentage relative to the wound closure in the control (PBS) group. Three areas of each wound (6 wounds/treatment) were quantified. Data are presented as mean ± SD (*n* = 6). *** *p* < 0.005.

Enhanced migration of LX-2 cells is often associated with the progression of fibrosis [45]. To evaluate anti-fibrotic activity of AM exosomes, we also assessed the migration of LX-2 cells using a scratch wound assay. As shown in Figure 5 and Supplemental Figure S2, the presence of exosomes reduced the migration of LX-2 by about 20% (AM/N) and 26% (AM/H). This result suggested that AM exosomes carry anti-fibrotic activity to inhibit the migration of LX-2 cells.

### 2.5. Characterization of Exosomes Released from In Vitro Cultured Cells Isolated from Amniotic Membrane

**Figure 6:**
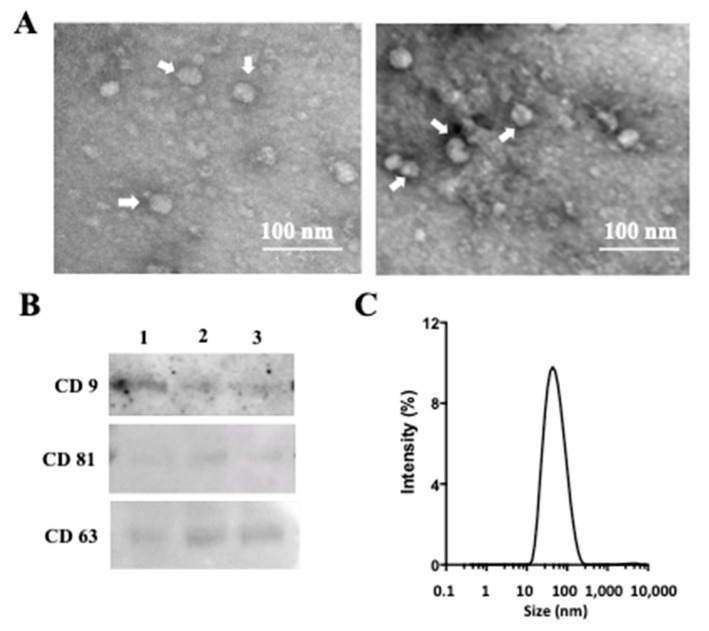
Characterization of exosomes isolated from AM-stromal cells. Exosomes were isolated from conditioned media prepared from AM-stromal cells. Exosomes were examined by transmission electron microscopy (**A**). Representative exosomes are indicated by white arrows. Scale bar = 100 nm. The presence of tetraspanins in exosomes was determined by SDS-PAGE followed by Western blot using antibodies against human CD9, CD63, and CD81 (**B**). Samples in Lane 1–3 were from different isolation batches. The particle sizes of exosomes were analyzed using dynamic light scattering. The size distributions were graphed against the percentage of intensity (**C**).

The exosomes isolated from AM tissues showed anti-fibrotic activity, suggesting that exosomes from tissue may be a source for the anti-fibrotic activity of AM-conditioned media reported previously [21]. In vitro cultured stromal cells isolated from AM have been shown to produce exosomes [36]. To understand if the exosomes isolated from the AM-tissue-conditioned media are the same as the exosomes released from AM stromal cells cultured in vitro, stromal cells were isolated from AM tissues, cultured, and expanded in vitro. Conditioned media were prepared from the stromal cells (P2). The exosomes were isolated from the cell-conditioned media following the same protocol used for the isolation of exosomes from AM tissue-conditioned media. The yield of exosomes was about 94 µg/10^6^ cells. As shown Figure 6A, spherical exosomes were detected by TEM (indicated by white arrows). The presence of tetraspanins was detected in AM-stromal-cell-produced exosomes (Figure 6B). However, in comparison with exosomes released from AM tissue (Figure 1A), the exosomes from cultured AM-stromal cells were smaller in size. Consistently with the TEM observation, while AM-tissue-released exosomes had an average size of about 75 nm (Figure 1C), exosomes released by cultured cells showed a single peak population with an average size of about 36 nm (Figure 6C).

### 2.6. The Effects of AM-Stromal Cell Released Exosomes on the Expression of Fibrotic Markers in LX-2 Cells

**Figure 7:**
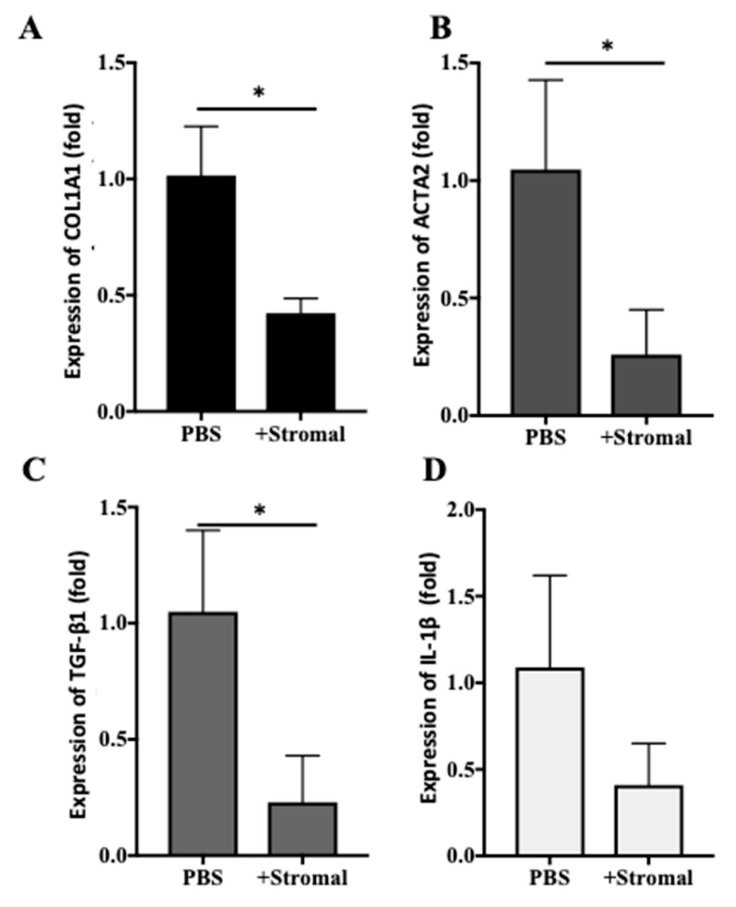
Effect of stromal cell exosomes on the expression of fibrotic markers in LX-2 cells. Non-activated LX-2 cells were cultured in the presence (+Stromal) or absence (PBS) of exosomes isolated from stromal cells for 2 days. Total RNA was isolated from LX-2 cells, and the relative expression of COL1A1 (**A**), ACTA2 (**B**), TGFβ1 (**C**), or IL-1β (**D**) was quantified by qPCR. The relative expression of each gene in the presence of exosomes was normalized to that of control (PBS), which was set as 1. Data are presented as mean ± SD (*n* = 3). * *p* < 0.05.

To evaluate whether the exosomes released by AM-stromal cells have anti-fibrotic activity, the expression of pro-fibrotic markers in LX-2 cells was evaluated in the presence or absence of exosomes (Figure 7). The expression of COL1A1, ACTA2, or TGF-β1 was reduced by the presence of exosomes (Figure 7A–C). The effect of stromal cell-derived exosomes on the expression of IL-1β was less prominent (Figure 7D). These results suggest that, similar to AM tissue-derived exosomes, stromal cell-derived exosomes also had an inhibitory effect on the expression of pro-fibrotic genes in LX-2.

## 3. Discussion

The clinical successes of amniotic membrane (AM) in treating chronic wounds have led to propositions of additional therapeutic applications of AM. Among many chronic disease conditions, fibrosis has been considered as a wound healing process gone awry [50]. Fibrosis of tissue/organ results in the dysfunction of such tissue/organ and eventually the loss of functions of key organs. Currently, there is no therapy available to reverse fibrotic processes [51]. The current treatments for pulmonary fibrosis mostly are drugs targeting signal transduction pathways involved in fibrosis to prevent the excess deposition of extracellular matrix [52,53]. The potential of AM in treating fibrotic diseases has been studied in animal models [16,17,18]. The anti-inflammatory and anti-fibrotic activities of AM have also been demonstrated in vitro [21,54]. Epithelial and stromal cells isolated from AM tissues have demonstrated anti-inflammatory and anti-fibrotic activities in various experimental systems [19,20,55]. Soluble factors have been attributed to these observed activities. In addition to growth factors and cytokines released by AM-derived cells, exosomes released by in vitro cultured cells have also been shown to carry out anti-fibrotic effects on target cells [42,43,44]. While there are numerous reports on the exosomes released by in vitro cultured AM-derived cells, the isolation and characterization of exosomes released by intact viable AM tissues has not been subject to investigation. Our previous study showed that conditioned medium from viable AM has anti-fibrotic activity [21]. One aim of this study is to understand whether exosomes released from AM tissues are a contributing factor to the anti-fibrotic activity of AM. Vesicles enriched at 100,000× *g* centrifugation from AM tissue conditioned medium showed prominent tetraspanins and characteristic nano size as observed in exosomes (Figure 1). In order to compare the tissue-released exosomes with in vitro cell-released exosomes, stromal cells were isolated from AM tissues and cultured in vitro (Figure 6). Cultured-stromal-cell-released exosomes have an average size of about 40 nm. On the other hand, exosomes isolated from AM tissues were larger in size (~75 nm on average). It is known that the production and characteristics of exosomes may be influenced by the physiological state of cells [23,30]. It is plausible that the AM cells in their native tissue microenvironment selectively produce and release larger-size exosomes. Our preliminary results also revealed that the average size of exosomes isolated from in vitro cultured cells may be influenced by passage number (data not shown). While the influence of isolation methods on the size distribution of exosomes has been reported [56], the passage number or population doubling levels (PDL) of cultured cells on the size of exosomes has not been addressed. The compositions of these three types of exosomes and the effect of PDL on exosomes released by in vitro cultured cells will be studied to further address the similarity and differences among tissue- and cultured-cell-released exosomes.

To assess whether exosomes released from AM tissue have anti-fibrotic activity, the effects of tissue exosomes on the growth, deposition of extracellular matrix, and expressions of fibrotic genes in hepatic stellate LX-2 cells were evaluated in vitro. LX-2 cells are the most widely used in vitro cell model to study hepatic fibrosis [8,45]. It has been reported that exosomes produced by human umbilical cord mesenchymal stem cells reduced the proliferation of LX-2 cells [57]. However, we did not observe an inhibitory effect of tissue-released exosomes on the proliferation of LX-2 cells at the concentrations we tested (Figure 2A,C and Supplemental Figure S1). On the other hand, when LX-2 cells were activated by TGF-β1, the presence of exosomes showed an inhibitory effect on the growth of LX-2 cells (Figure 2B,D). The reduced cell growth may be partially explained by the reduced proliferation of activated LX-2 cells when treated with exosomes (Supplemental Figure S1).

The increased migration of LX-2 is often associated with fibrotic responses [45]. In the presence of tissue-released exosomes, the migration of LX-2 was reduced (Figure 5). Moreover, the effect of tissue-released exosomes on the expression of fibrotic markers in LX-2 cells was evaluated by qPCR. We showed a decrease in gene and protein expression of Col 1 in the presence of exosomes (Figure 3A and Figure 4A). Over-expression of Col 1 and alpha-smooth actin (αSMA) are associated with the progression of fibrotic processes [58,59]. The reduction in the expression of Col 1 and αSMA by tissue-released exosomes is likely to attenuate the fibrotic phenotype of LX-2 cells. More interestingly, the presence of exosomes significantly reduced the TGF-β1 stimulated expression of fibrotic markers and suggested that exosomes counteracted the pro-fibrotic effect of TGF-β1 on LX-2 cells. Altogether, our results suggested that exosomes released from AM tissue show anti-fibrotic activity. However, LX-2 cells, as an in vitro fibrosis model, has limitations due to the lack of microenvironment and other cell types found in the in vivo fibrosis process. The anti-fibrotic effect of AM-derived exosomes on bleomycin-induced liver fibrosis is currently under investigation in a mouse model.

The cargo of exosomes is determined by the origin, microenvironment, and physiological state of cells [60]. The compositions of exosomal proteins, nucleic acids, and lipids are reported to be diverse in the literature, and such information is regularly updated in exosome databases [61,62]. Numerous studies have identified exosomal microRNAs as the modulators of wound healing and fibrosis progression. For example, miR-21 in human fibrocyte-derived exosomes directly regulate type I collagen expression and protein deposition [63]. miRNAs such as miR-21, miR-23a, miR-125b, and miR-145 have been identified in exosomes secreted from umbilical-cord-derived mesenchymal stem cells. These miRNAs inhibit the differentiation of myofibroblasts by modulating the TGF-β/SMAD2 pathway [64]. Exosomes from embryonic stem cells are enriched with miR-200a, which mediates the rejuvenation of senescent endothelial cells by regulating the expression of Keap1 and Nrf2 [65]. The identities of miRNA and their roles in anti-fibrotic activity of AM-derived exosomes will be investigated in our next study.

It has been shown that the composition and activity of paracrine components (including secretion of exosomes) by tissues or cultured cells are regulated by signaling pathways such as RAP1/NFκB [66,67]. Inhibition of RAP1/NFκB activation results in the production of pro-regenerative paracrine components [68]. Therefore, the involvement of such signaling pathways in the production of tissue-derived or cultured cell-derived exosomes warrants future investigation.

Hypoxic condition has been shown to stimulate the production of exosomes from in vitro cultured cells [23,30]. In this study, we also showed that AM tissues respond to hypoxic conditions and release exosomes with higher levels of tetraspanins compared with AM tissues under normoxic conditions (Figure 1). Furthermore, the exosomes isolated from hypoxic conditions showed a stronger anti-fibrotic activity than those isolated from normoxic conditions. Wound environment and fibrotic tissues are often associated with hypoxia [69,70,71]. When viable AM tissues are applied at the wound site, the enhanced production and release of active exosomes from AM tissues may contribute to the observed clinical benefit of viable AM tissue [72].

Application of AM tissue directly onto fibrotic liver has been studied in rodent models, and the reduction in liver fibrosis has been reported [15,16,17,18]. While the application of amniotic membrane to fibrotic organs of human patients is challenging, exosomes from AM tissues may be a more attractive alternative. In fact, exosomes from in vitro cultured bone-marrow-derived mesenchymal stem cells, AM-derived epithelial cells, and AM-derived stromal cells have been explored as the emerging cell-free therapy for fibrotic diseases [46]. However, the tissue-released exosomes have not been explored. Our results demonstrated that AM tissues release functional exosomes. By testing exosomes isolated from different donors individually, we observed similar characteristics and comparable effects on LX-2 cells (data not shown). Due to the immune privilege of AM tissue and low immunogenicity of exosomes [73,74], exosomes isolated from AM tissues from multiple donors may readily be combined and used as a cell-free therapy.

## 4. Materials and Methods

### 4.1. Materials

Human hepatic stellate cell line (LX-2) was purchased from Millipore/Sigma (Scc064, Burlington, MA, USA). DMEM (low glucose or high) was purchased from HyClone (Logan, UT, USA). Fetal bovine serum (Hi-FBS), exosomes-depleted FBS, 100× antibiotic/antimycotic solution were from Gibco (Gaithersburg, MD, USA). LX-2 cells were cultured in DMEM (high glucose) containing 2% FBS and 1× antibiotic/antimycotic following the vendor’s instruction. Recombinant human TGF-β1 (Cat# 100–21) was purchased from PeproTech (Rocky Hill, NJ, USA). Antibodies, anti-CD9 (sc-13118), anti-CD81 (sc-7637), anti-CD63 (sc-5275) were from Santa Cruz Biotechnology (Dallas, Texas). Anti-human type I collagen (Col1) antibodies (ab34710) were from Abcam (Cambridge, MA, USA). Anti-beta-actin: THE^TM^ beta actin mAb antibodies (A00702) (100 µg/mL) were from GenScript (Piscataway, NJ). For qPCR analysis, the QuantiTect primers, Hs_ACTB_1_SG (QT00095431), Hs_COL1A1_1 (QT00037793), Hs_ACTA2_1 (alpha smooth muscle actin) (QT00088102), Hs_TGFB1 (QT00000728), Hs_TGFB3 (QT00001302), Hs_HGF(hepatocyte growth factor) (QT01758988), and Hs_IL1B (QT00021385) were purchased from Qiagen (Germantown, MD, USA).

### 4.2. Preparation of Conditioned Media from Human Amniotic Membranes under Normal and Hypoxic Conditions

The procurement of human full-term placenta and ethical statement were provided by the National Disease Research Interchange (Philadelphia, PA, USA) and Cord Blood America, Inc. (Las Vegas, NV, USA). Tissues were collected from eligible donors after obtaining written, informed consent [75]. The isolation and processing of human amniotic membranes (AM) were carried out as described [54]. Four pieces of 5 × 5 cm of AM samples from each of four different donors were conditioned as described [15]. Briefly, four pieces of AM samples (total 100 cm^2^) were submerged and free-floating in 12 mL of DMEM medium containing 5% exosomes-free FBS in a 10 cm diameter petri dish. For AM from each donor, two sets of samples were prepared. One set of tissues were conditioned in an incubator at 37 °C, 5% CO_2_, and 95% humidity with 20% O_2_ (normoxia), the other set of tissues were conditioned in an incubator at 37 °C, 5% CO_2_, and 95% with 2% O_2_ incubator (hypoxia). After conditioning for 48 h, the tissue pieces were removed and the conditioned media collected, appropriately labeled, and stored at −80 °C till exosome isolation.

### 4.3. Isolation of Stromal Cells from AM

Fresh AM tissue was washed in PBS and then cut into small pieces. The tissues were then digested for 7 min using 0.25% Trypsin (Gibco, Gaithersburg, MD, USA) in a 37 °C water bath with gentle agitation, allowing for epithelial cells to detach from the membrane surface. Trypsin solution was then neutralized by 10% exosomes-depleted FBS. After removing the epithelial layer, the tissue pieces were then washed three times in excess HBSS and subsequently digested with 20 mg collagenase in 200 mL HBSS for 30 min with gentle agitation in a 37 °C water bath. After centrifugation, the stromal cell pellets were collected and washed twice with HBSS. The cells (5 million/mL) were then cryopreserved in liquid nitrogen for future use. The multi-potency of the isolated stromal cells was verified by osteogenic and adipogenic differentiation using standard differentiation protocols (data not shown).

### 4.4. Preparation of Conditioned Media from AM-Stromal Cells Cultured In Vitro

Stromal cells isolated from AM tissues (AM-stromal cells, P0) were cultured and expanded in DMEM (low glucose) medium + 10% Hi FBS + antibiotic/antimycotic. AM-stromal cells (P2) were cultured in a 10 cm diameter tissue culture dish to about 70% confluence. The culture medium was removed and 9 mL of DMEM (low glucose) medium containing 5% exosomes-depleted FBS was added to each dish and cultured for another 48 h. The supernatant from 12 dishes of cells was collected and stored at −80 °C till use.

### 4.5. Isolation of Exosomes from AM-Conditioned Media or Stromal-Cells-Conditioned Media

Sequential ultracentrifugation method was used to isolate the exosomes from conditioned media as described [30] with modifications. Conditioned media were thawed and centrifuged at 1000× *g* at 4 °C for 25 min using Beckman Coulter Allegra 6R Centrifuge. Supernatant was collected and transferred to a polypropylene Quick-Seal centrifuge tube (Beckman Coulter, Brea, CA, USA) and centrifuged at 10,000× *g* at 4 °C for 60 min in a Beckman Coulter Optima L-90K Ultracentrifuge with Type −70.1 Ti rotor. Supernatant was collected and transferred to a new Quick-Seal centrifuge tube and centrifuged at 100,000× *g* at 4 °C for 3 h. Exosomes were washed with 5 mL of phosphate-buffered saline (PBS) once and resuspended in PBS (1 mL/tube for AM-conditioned media or 0.4 mL/tube for stromal cell-conditioned media). The resuspended exosomes were filtered through 0.22 µm syringe filter. Filtered exosomes were quantified using Pierce^TM^ BCA protein assay kit (ThermoFisher Scientific, Waltham, MA, USA) following manufacturer’s instruction. Exosomes were then aliquoted and stored at −80 °C till use. The exosomes from four different donors were combined for all experiments.

### 4.6. Transmission Electron Microscopy (TEM) Analysis of Exosomes

Exosomes in PBS were diluted using pure water at 1:5. Five microliters of diluted exosomes were dropped onto a formvar-carbon coated 300-mesh copper grid (Electron Microscopy Sciences, Hatfield, PA, USA) and left to dry at room temperature for 2 min. The exosome samples were stained using Uranyl acetate 2% solution (Electron Microscopy Sciences, Hatfield, PA, USA). The grids were dried at room temperature (RT) and then viewed in a Philips CM12 electron microscope with AMT-XR11 digital camera.

### 4.7. Particle Size Analysis of Exosomes

Dynamic light scattering analysis (DLS) was used to determine the particle size distribution, and 0.5 mL of exosomes in PBS was added to a cuvette and analyzed using Zetasizer Nano Series (Malvern Instruments, Worcestershire, UK). Data were analyzed using Zetasizer Nano Series (Nano-S) Software.

### 4.8. Western Blot Analysis

For analysis of the exosomes, the same volume of 2× LDS (lithium dodecyl sulfate) sample buffer (GenScript, Piscataway, NJ) was added to 1 μg (stromal cell-derived) or 2 μg (AM-tissue derived) of exosomes in PBS in a 1.5 mL microfuge tube. For analysis of type I collagen (Col1), cells in 48-well plates were lysed in 100 μL of 2× LDS sample buffers. Exosome samples or cell lysates were boiled and cooled down and separated using SDS-PAGE on gradient (4–20%) Precast Gels (GenScript, Piscataway, NJ, USA) and transferred to the nitrocellulose membrane using Mini-PROTEAN^®^ Gel Transfer Device (Bio-Rad, Hercules, CA, USA). Membranes were blocked in 5% nonfat milk in 1x Tris-buffered saline-Tween 20 (TBS-T) buffer for 2 h at RT and then incubated overnight with primary antibody at RT with gentle shaking. The membrane was incubated with a suitable secondary antibody conjugated with horseradish peroxidase (HRP) and visualized using KwikQuant^TM^ Ultra Digital-ECLTM Substrate Solution (R1002) and KwikQuant^TM^ imager (Kindle Biosciences, Greenwich, CT, USA). The following anti-human antibodies were used as primary antibodies: anti-CD9 (1:200), anti-CD63 (1:100), anti-CD81 (1:200), anti-Col1 (1:2000), and anti-beta-actin (1:10,000). Goat-anti-mouse IgG-HRP (HAF007, R&D system, Minneapolis, MN, USA) at 1:10,000 or Goat-anti-rabbit IgG-HRP (G21234, ThermoFisher Scientific, Carlsbad, CA, USA) at 1:10,000 was used as secondary antibody.

### 4.9. Measurement of the Growth of Non-Activated or Activated LX-2 Cells

LX-2 cells in DMEM complete medium (DMEM (high glucose) containing 2% FBS and antibiotic/antimycotic) were seeded at 2 × 10^4^/well in a 48-well plate and cultured overnight at 37 °C with 5% CO_2_ and 95% humidity. On Day 0, one set of the LX-2 cells were activated by treating with 4 ng/mL TGF-β1 in DMEM complete medium for 3 h. Exosomes were added to activated or non-activated LX-2 cells in DMEM complete medium and incubated for 48 h. As a control, PBS (the solution used to resuspend exosomes after isolation) was added to LX-2 cells at the same volume as that of exosomes. The viability of cells was measured by alamarBlue assay. The percentage of growth of LX-2 in each well was expressed as fluorescent intensity on Day 2/ fluorescent intensity on Day 0 × 100%.

### 4.10. Scratch Wound Migration Assay

LX-2 cells were seeded at 1 × 10^5^/well in 24-well plates and cultured in DMEM complete medium to confluence. A scratch wound crossing through the cell layer in each well was created using a 20 µL pipet tip. Wells with scratched cell layers were rinsed once with PBS. Phase-contrast images of wounds were taken at time 0 for all conditions. Fresh medium with or without exosomes (5 μg/mL) was added to cells and incubated at 37 °C for 24 h. Phase-contrast images of wounds were taken at 24 h for all conditions. Image J software was used to measure the wound area. The migrated area = Wound area at T_0h_—Wound area at T_24h_. The relative migration = Migrated area with exosome/Migrated area with PBS × 100%.

### 4.11. Immunofluorescence Staining and Microscopy

In order to visualize the collagen matrix produced by LX-2 cells, 2 × 10^4^/well of cells were cultured in 48-well plates in the presence or absence of AM exosomes for 5 days with changing of medium every two days. The cells were fixed with 4% paraformaldehyde (PFA), permeabilized with 0.5% Triton ×100 and blocked with 5% FBS and 1% BSA in PBS prior to incubation with primary antibody anti-Col1 (ab34710) at 1:150 at 4 °C overnight. Goat-anti-rabbit IgG-Alexa488 at 1:10,000 and Hoechst 34580 (ThermoFisher Scientific, Carlsbad, CA, USA) at 1:10,000 were used to detect the primary antibody and DNA. Stained samples were imaged using an epi-fluorescent microscope (Zeiss Axio Observer D1, Jena, Germany). Images were captured using Axiovision software and Zeiss AxioCam MRm camera.

### 4.12. Quantification of the Relative Expression of mRNA by qPCR

The quantification of the relative expression of fibrotic markers by qPCR was performed as previously described [21]. Briefly, LX-2 cells or activated LX-2 cells were cultured in the presence or absence of exosomes for 48 h. The cells were then lysed in 0.2 mL of RNA lysis buffer (Promega, Durham, NC, USA). Total RNA from these lysates was purified using SV 96 Total RNA Isolation System (Promega, Madisin, WI, USA). RNA concentration and purity were measured using TECAN Spark Nano plate (TECAN, Morrisville, NC, USA). cDNA preparation and qPCR were performed as described [76]. The primers used for qPCR were described in Materials. Each sample was run in duplicate. After the run was completed, a second derivative analysis was performed using the raw data to determine the mean Cp (Crossing point-PCR-cycle) for each sample. For each gene expression, expression of beta-actin served as an internal control. Relative mRNA expression was determined by Pfaffl analysis (EΔCp target/EΔCp reference), in which primer efficiency E = 10^(−1/slope) and ΔCp = mean Cp of sample − mean Cp of the cells treated with PBS (Control).

### 4.13. Statistical Analysis

Statistical analysis was performed as described [21]. Each independent experiment contained 3 or more biological repeat samples (*n* ≥ 3), and data are presented as the mean ± standard deviation. Results shown are representative of at least two independent experiments. One-way ANOVA with a Tukey’s multiple comparisons test was performed to determine statistical significance using GraphPad Prism, GraphPad Software (La Jolla, CA, USA, www.graphpad.com (accessed on 24 January 2021), version 7.0d for Mac OS X, 16 November 2017) for all quantitative data. Differences were considered significant at a *p*-value of < 0.05.

## 5. Conclusions

Viable amniotic membrane (AM) releases functional exosomes, which contribute to the anti-fibrotic activity of AM. Anti-fibrotic effects of tissue-released exosomes on LX-2 suggest the potential application of AM-tissue-released exosomes as a cell-free therapy for fibrotic diseases.

## Data Availability

Data is contained within the article and supplementary material.

## Supplementary Materials

The following are available online at https://www.mdpi.com/1422-0067/22/4/2055/s1, Figure S1: Detection the proliferation of LX-2 cells by EdU staining; Figure S2: Migration of LX-2 cells in the presence of exosomes.
