# Supplementary material for: Exosomes Secreted from Amniotic Membrane Contribute to Its Anti-Fibrotic Activity
Source: Int J Mol Sci. 2021 Feb 19;22(4):2055. doi: 10.3390/ijms22042055 (PMC7922650; doi:10.3390/ijms22042055)
Supplement: Supplementary file 1 [file ijms-22-02055-s001.zip › ijms-1104133-supplementary.pptx]

## Slide 1
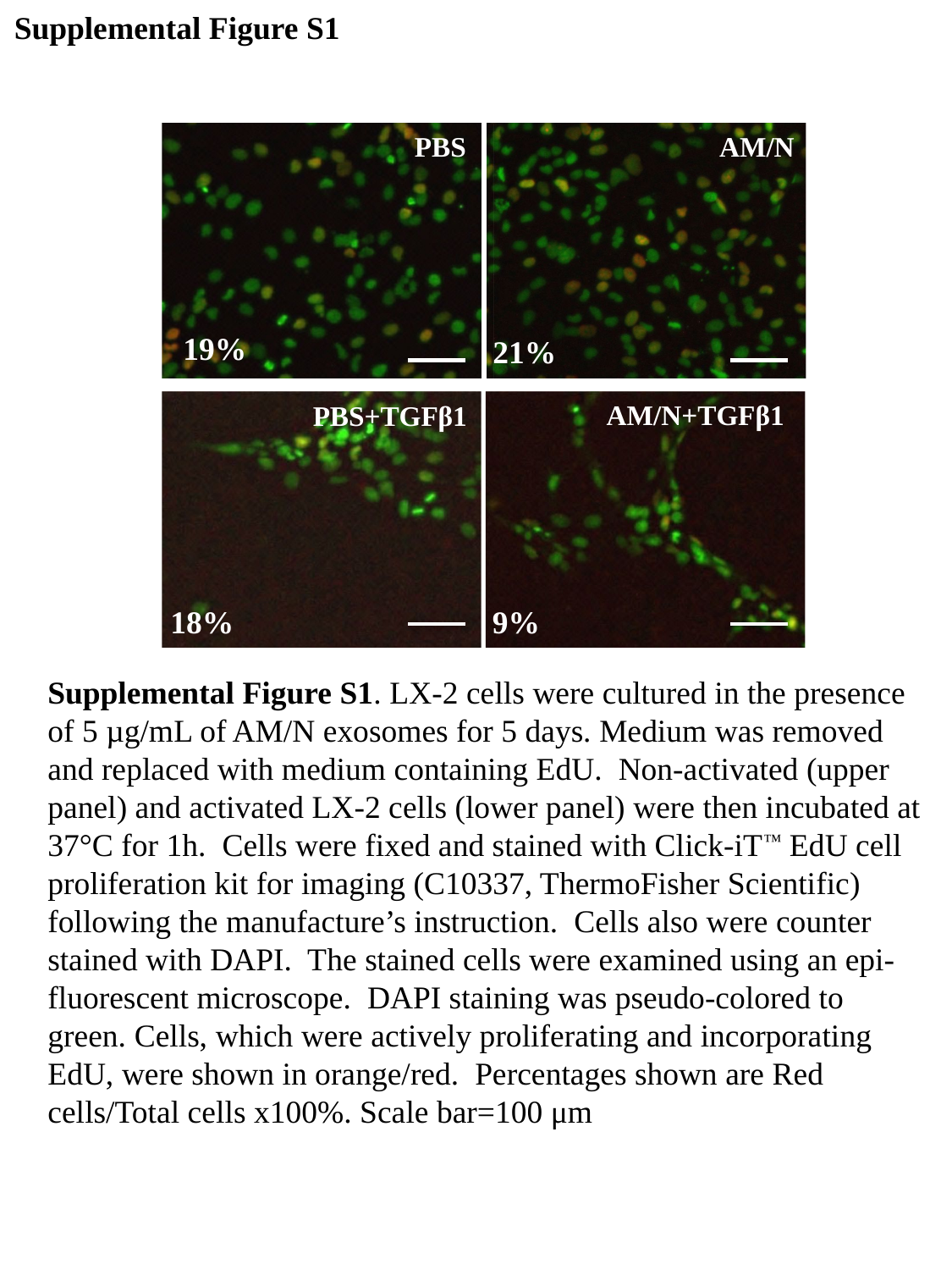

Supplemental Figure S1
PBS
AM/N
19%
21%
AM/N+TGFβ1
PBS+TGFβ1
18%
9%
Supplemental Figure S1. LX-2 cells were cultured in the presence of 5 µg/mL of AM/N exosomes for 5 days. Medium was removed and replaced with medium containing EdU. Non-activated (upper panel) and activated LX-2 cells (lower panel) were then incubated at 37°C for 1h. Cells were fixed and stained with Click-iT™ EdU cell proliferation kit for imaging (C10337, ThermoFisher Scientific) following the manufacture’s instruction. Cells also were counter stained with DAPI. The stained cells were examined using an epi-fluorescent microscope. DAPI staining was pseudo-colored to green. Cells, which were actively proliferating and incorporating EdU, were shown in orange/red. Percentages shown are Red cells/Total cells x100%. Scale bar=100 μm

## Slide 2
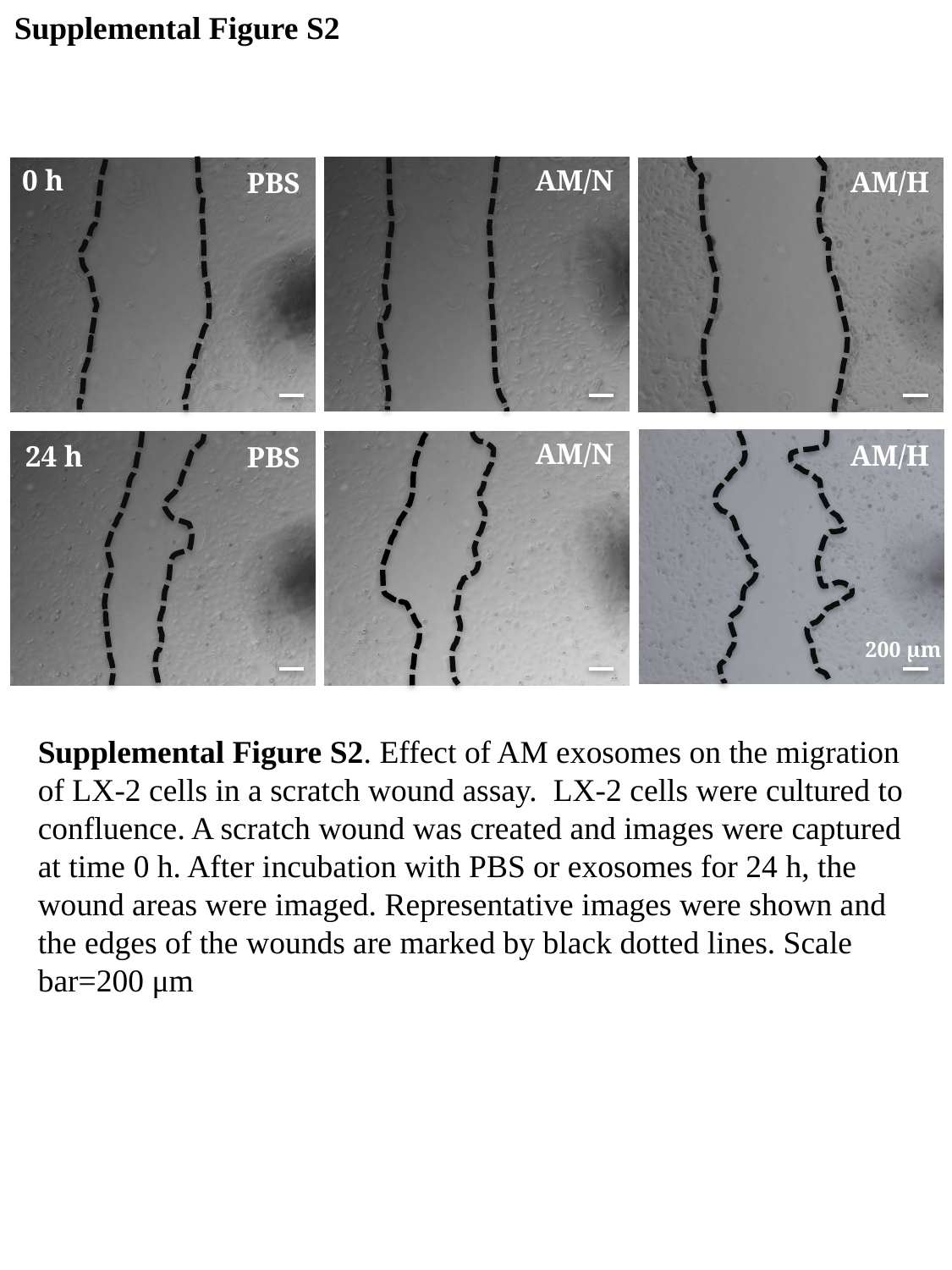

Supplemental Figure S2
0 h
AM/N
AM/H
PBS
AM/N
AM/H
24 h
PBS
200 μm
Supplemental Figure S2. Effect of AM exosomes on the migration of LX-2 cells in a scratch wound assay. LX-2 cells were cultured to confluence. A scratch wound was created and images were captured at time 0 h. After incubation with PBS or exosomes for 24 h, the wound areas were imaged. Representative images were shown and the edges of the wounds are marked by black dotted lines. Scale bar=200 μm
